# Quantum and thermal noise in coupled non-Hermitian waveguide systems with different models of gain and loss

**DOI:** 10.1515/nanoph-2024-0512

**Published:** 2025-01-03

**Authors:** Osmery Hernández, Iñigo Liberal

**Affiliations:** Department of Electrical, Electronic and Communications Engineering, Public University of Navarre, 31006 Pamplona, Spain; Institute of Smart Cities, Public University of Navarre, 31006 Pamplona, Spain

**Keywords:** gain-loss compensation, non-Hermitian photonics, exceptional points, quantum noise, thermal noise, squeezing

## Abstract

Non-Hermitian (NH) photonic systems leverage gain and loss to open new directions for nanophotonic technologies. However, the quantum and thermal noise intrinsically associated with gain/loss affects the eigenvalue/eigenvector structure of NH systems, and thus the existence of exceptional points, as well as the practical noise performance of these systems. Here, we present a comparative analysis of the impact of different gain and loss mechanisms on the noise generated in gain–loss compensated NH waveguide systems. Our results highlight important differences in the eigenvalue/eigenvector structure, noise power, photon statistics and squeezing. At the same time, we identify some universal properties such as the occurrence of phase-transition points in parameter space and intriguing phenomena related to them, including coalescence of pairs of eigenvectors, gain–loss compensation, and linear scaling of the noise with the length of the waveguide. We believe that these results contribute to a better understanding of the impact of the gain/loss mechanism on the noise generated in NH systems.

## 1 Introduction

Non-Hermitian (NH) systems are ubiquitous among real physical systems since describing a system as an isolated entity is often impossible or inaccurate [1]. Since any external environment can either pump into or retract energy from a system, NH systems are usually associated with gain, loss, or a combination of both [2]. In addition, Hermitian operators have real eigenvalues, while the eigenvalues of NH operators are generally complex numbers. However, a reduced group of NH operators satisfying a weaker constraint than Hermiticity can still have a real spectrum in a given region in parameter space. These systems exhibit PT-symmetry [3], [4], or in other words, their Hamiltonian $\left(\hat{H}\right)$ commutes with the joint operation of parity $\left(\hat{P}\right)$ and time reversal $\left(\hat{T}\right)$ .

Photonics have been an instrumental platform in the demonstration of PT-symmetric systems, given the equivalence between the Schrödinger equation and the paraxial wave approximation of Helmholtz equation, where the complex refractive index plays the role of the complex potential in the NH Hamiltonian [5], [6], [7]. Therefore, by engineering the refractive index through the appropriate combination of gain and loss, it is possible to obtain the real even and imaginary odd refractive index profiles characteristic of PT-symmetric systems. The transition between the regions where PT-symmetry is conserved, or unbroken phase, and the broken phase usually features spectral degeneracies known as exceptional points (EPs) [7], [8]. At these transition points, the eigenvalues coalesce, and so do the eigenvectors; thus the eigenspace is skewed. EP degeneracies have also been studied in the context of Liouvillian operators, scattering matrix approaches or using coupled mode theory [2].

PT-symmetry and EP degeneracies endow physical systems with intriguing non-trivial properties inaccessible to Hermitian systems, thus motivating intense research on the field in the last few years [2], [5], [8], [9], [10], [11], [12], [13], [14]. Examples of such counterintuitive effects include unidirectional reflectionless light propagation, and therefore, unidirectional invisibility [15], [16], [17], non-reciprocal light propagation [18], [19], loss-induced transparency [20], [21] and lasing [22], PT-symmetric lasers [23], [24] and CPA-lasers [25], [26], and chiral mode switching [27], to name a few. EPs have also been proposed to enhance the performance of sensors [28], [29]. Although the noise performance of EP sensors is the subject of current debate [30], [31].

In the quantum optics realm, systems featuring PT-symmetry and EPs have been reported to influence quantum interference [32], [33], [34], entanglement [35], [36], and decoherence [37], [38]. At the same time, it has been claimed that obtaining PT symmetry in quantum photonics systems combining loss and gain is not possible [39] due to the additional noise contribution of gain at the quantum level, which changes the underlying eigenvalue structure. However, this study only considered models for linear gain and loss mechanisms. Different alternatives have been explored in studies on quantum PT-symmetry and EPs trying to overcome the noise issues associated to linear or phase-insensitive amplification, most of them either rely on passive implementations or consider a non-Hermitian subsystem embedded in a large Hermitian system [6], [32]. Recently, the potential of phase-sensitive amplification and deamplification have been showcased [6], [31], demonstrating quadrature-PT symmetry and squeezing, what suggests that the model of gain and loss employed plays a critical role.

In this work, we present a general study on how the different quantum models of gain and loss impact the performance of Non-Hermitian photonic systems, and affect their underlying eigenvalue/eigenvector structure. To this end, we propose a theoretical framework to compute the quantum eigenmodes of the spatial evolution of coupled waveguides, which applies to different models of gain and loss, as well as all their possible combinations. We also clarify how such algebraic quantum eigenmodes can be measured with conventional measurement setups at the output of the waveguides. Our results provide an insightful perspective into how the nature of gain and loss mechanisms defines the nature of Non-Hermitian phase transitions, including their eigenvalue/eigenvector structure, the potential existence of EPs, the unusual scaling of quantum noise generation with the length of the waveguide, and the generation of squeezing in non-Hermitian systems. Our results also contribute to understanding the noise generated in gain–loss compensation systems, as a function of the models of gain and loss.

## 2 Theoretical framework

In this section we introduce a theoretical framework to model how the presence of gain and loss and their linear or parametric nature influence the evolution of quantum light states in photonic systems characterized by modes propagating along a given distance while exchanging excitations between them. For instance, that might be the case of a finite number of coupled waveguides or resonators. This framework not only provides an analytical solution to the evolution equation of the coupled modes, allowing for the computation of photon statistics, but also addresses intuitively the question of how to design the measurement setup to characterize the singular eigenvector/eigenvalue properties of non-Hermitian systems.

### 2.1 Spatial evolution in coupled non-Hermitian waveguides

**Figure 1:**
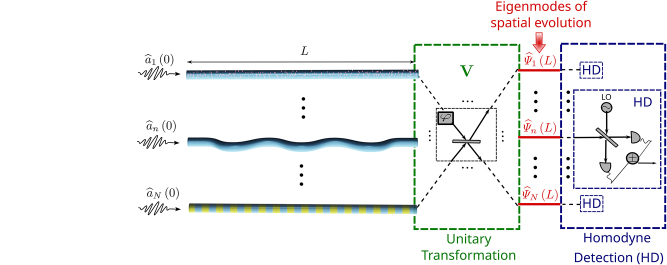
Schematic depiction of *N* interacting photonic waveguides with different classes of gain and loss. At the output of the waveguides, a unitary transformation induces a change of basis of the photonic modes, allowing for physical separation of the eigenmodes of the spatial evolution. Finally, homodyne detection enables the characterization of the photon statistics of the eigenoperators of the waveguide system.

As schematically depicted in Figure 1, let us start by mathematically modeling a photonic system where a finite set of photonic quantum modes evolve along a given distance and might couple with each other while propagating. Provided that the systems considered are in general non-Hermitian, they might exhibit gain and loss, either linear or parametric. The propagation or spatial evolution of the modes in these photonic systems can be described through the differential equation

(1) $$\partial_{z}\hat{a}\left(z\right)=M\;\hat{a}\left(z\right)+\hat{F}\left(z\right)$$

where $\hat{a}\left(z\right)={\left[{\hat{a}}_{1}\left(z\right),\ldots ,{\hat{a}}_{N}\left(z\right),{\hat{a}}_{1}^{†}\left(z\right),\ldots ,{\hat{a}}_{N}^{†}\left(z\right)\right]}^{T}$ is the column vector of the photonic annihilation $\left({\hat{a}}_{m}\left(z\right)\right)$ and creation $\left({\hat{a}}_{m}^{†}\left(z\right)\right)$ operators satisfying bosonic commutation relations $\left(\left[{\hat{a}}_{m}\left(z\right),{\hat{a}}_{n}\left(z^{\prime }\right)\right],=,0,\;,\text{and},\;,\left[{\hat{a}}_{m}\left(z\right),{\hat{a}}_{n}^{†}\left(z^{\prime }\right)\right],=,\delta ,\left(z-z^{\prime }\right),\;,\delta_{mn}\right)$ , while $\hat{F}\left(z\right)={\left[{\hat{F}}_{1}\left(z\right),\ldots ,{\hat{F}}_{N}\left(z\right),{\hat{F}}_{1}^{†}\left(z\right),\ldots ,{\hat{F}}_{N}^{†}\left(z\right)\right]}^{T}$ contains operators representing Langevin noise sources [40], [41], and $M\in C^{2N\times 2N}$ is the spatial evolution matrix. It is important to point out that, according to our notation, **M** = −*i* **H** with respect to the Hamiltonian description of NH waveguides, with the corresponding consequences in the physical interpretation of real and complex eigenvalues.

The structure of the noise vector $\hat{F}\left(z\right)$ in (1) will depend on the system under study: if there is gain or loss associated with the corresponding photonic mode and its linear or parametric nature. On the one hand, describing gain and loss through linear models involves the presence of thermal noise sources to preserve the bosonic commutation relations. These noise sources can be represented through bosonic creation $\left({\hat{f}}_{n}^{†}\left(z\right)\right)$ or destruction $\left({\hat{f}}_{n}\left(z\right)\right)$ noise operators, respectively, with their associated commutator $\left[f_{m}\left(z\right),f_{n}^{†}\left(z^{\prime }\right)\right]=\delta \left(z-z^{\prime }\right)\delta_{mn}$ . Therefore, for a photonic mode *m* experiencing linear loss *α*, we can define the Langevin noise vector component ${\hat{F}}_{m}$ in terms of a bosonic noise operator: ${\hat{F}}_{m}\left(z\right)=\sqrt{2\alpha }\;{\hat{f}}_{m}\left(z\right)$ , with commutation relations $\left[{\hat{F}}_{m}\left(z\right),{\hat{F}}_{n}^{†}\left(z^{\prime }\right)\right]=2\alpha \;\delta \left(z-z^{\prime }\right)\delta_{mn}$ [40], [42]. Similarly, ${\hat{F}}_{m}\left(z\right)=\sqrt{2g}\;{\hat{f}}_{m}^{†}\left(z\right)$ for a mode subject to amplification with linear gain *g*, and the associated commutator is given by $\left[{\hat{F}}_{m}\left(z\right),{\hat{F}}_{n}^{†}\left(z^{\prime }\right)\right]=-2g\;\delta \left(z-z^{\prime }\right)\delta_{mn}$ [40], [42].

On the other hand, gain and loss can also arise from nonlinear or parametric phenomena leading to squeezing transformations and mixing creation and destruction photonic operators. Squeezing transformations are unitary operations that guarantee the conservation of the bosonic commutation relations without the need to resort to additional noise sources. Modeling both phenomena through the same process is not an arbitrary decision; it is justified because a parametric gain can also be regarded as a parametric loss mechanism, depending on the quadrature component we observe, as one of them is amplified while the other is attenuated given the phase-sensitive nature of the process [40]. Again, no additional noise sources are required for parametric gain/loss, and their behavior is fully contained within the matrix **M**.

### 2.2 Eigenoperators of spatial evolution

In general, calculating photon statistics at the output of the waveguides can be a complicated task. However, the analysis simplifies for specific linear combinations of the waveguide modes for which their spatial evolution decouple. Importantly, such modes can be identified even for non-diagonalizable evolution matrices **M**, as is typically the situation at the phase transition of NH systems, where eigenvectors coalesce. To show how this is the case, we introduce a new operator given by a linear combination of waveguide mode operators ${\hat{\Psi }}_{n}\left(z\right)=\overset{2N}{\underset{m=1}{\sum }}c_{m}{\hat{a}}_{m}\left(z\right)$ . Following Eq. (1), the spatial evolution of ${\hat{\Psi }}_{n}\left(z\right)$ is given by

(2) $$\partial_{z}{\hat{\Psi }}_{n}=\overset{2N}{\underset{n=1}{\sum }}\left(\overset{2N}{\underset{m=1}{\sum }}c_{m}M_{mn}\right){\hat{a}}_{n}\left(z\right)+\overset{2N}{\underset{m=1}{\sum }}c_{m}{\hat{F}}_{m}\left(z\right)$$

From Eq. (2) it is apparent that if such a linear combination corresponds to a left eigenvector of the dynamic matrix **M**, i.e., $\overset{2N}{\underset{m=1}{\sum }}c_{m}M_{mn}=\lambda_{n}c_{n}$ , then

(3) $$\partial_{z}{\hat{\Psi }}_{n}\left(z\right)=\lambda_{n}{\hat{\Psi }}_{n}\left(z\right)+{\hat{\zeta }}_{n}\left(z\right)$$

where ${\hat{\zeta }}_{n}\left(z\right)=\overset{2N}{\underset{m=1}{\sum }}c_{m}{\hat{F}}_{m}\left(z\right)$ , and we can clearly distinguish that the evolution of the eigenoperators defined through the left eigenvectors of the dynamic matrix **M** is not interacting, and can be computed independently. Accordingly, it is possible to obtain the eigenoperator at the output of the waveguide upon direct integration:

(4) $${\hat{\Psi }}_{n}\left(L\right)={\hat{\Psi }}_{n}\left(0\right)\;e^{\lambda_{n}L}+e^{\lambda_{n}L}\int_{0}^{L}dz^{\prime }e^{-\lambda_{n}z^{\prime }}{\hat{\zeta }}_{n}\left(z^{\prime }\right)$$

Equation (4) states that the eigenoperator at any given distance *L* on the waveguide can be computed from the knowledge of the eigenoperator at the start of the waveguide, i.e., at *z* = 0 and its associated noise component.

The most appealing reading of this result is that for any given photonic quantum system, if we perform a unitary transformation that creates adequate linear combinations of the original bosonic modes, the associated evolution would be easily computed from the knowledge of the initial conditions in the system. We note that even if the matrix **M** is not diagonalizable, and their eigenvectors do not span the complete $C^{2N}$ space, individual eigenmodes can nevertheless be physically separated with a unitary matrix **V** that does span the complete $C^{2N}$ space, and contains the eigenmode as a member of its basis. Therefore, the photon statistics of individual eigenoperators can be measured in practice even at the degeneracy points of NH systems. As previously pointed out, we must compute the left eigenvectors of the evolution matrix **M**, or, equivalently, the right eigenvectors of the transposed evolution matrix **M** ^*T*^.

### 2.3 Diagonalizable evolution matrices

Naturally, the result showcased in Eq. (4) can be more easily obtained for the particular case that the evolution matrix is diagonalizable, i.e., **M** = **V** ^**−1**^ **DV**, where **V** is an invertible matrix whose columns are eigenvectors of the system and constitute a complete basis of $C^{2N}$ , while the diagonal entries in the diagonal matrix **D** corresponds to the associated eigenvalues *λ*_*n*_. In this case, one can simply change the basis of the photonic and noise operators to $\hat{\Psi }\left(z\right)=V\hat{a}\left(z\right)$ and $\hat{\zeta }\left(z\right)=V\hat{F}\left(z\right)$ , respectively, so that their evolution can be described through a diagonal matrix

(5) $$\partial_{z}\hat{\Psi }\left(z\right)=D\hat{\Psi }\left(z\right)+\hat{\zeta }\left(z\right)$$

Therefore, the photon statistics of the eigenoperators associated with left eigenstates of the system can be easily computed by separating such eigenoperators through an optical network implementing the matrix **V** (see Figure 1). The crucial difference between diagonalizable and non-diagonalizable evolution matrices is that when **M** is non-diagonalizable, as it is common in NH systems, not all the light exiting the waveguides can be described via the eigenoperators, since the associated eigenvectors do not span the entire $C^{2N}$ space. Despite this fact, the light and quantum noise associated with individual eigenoperators can be separated via unitary transformations, so that such algebraic singular eigenvectors/eigenvalues correspond with measurable photon statistics.

### 2.4 Symmetries of the eigenvalues, eigenvectors and eigenoperators

Next, we discuss the symmetries of the eigenvectors of the matrix **M**, which further clarify how to measure individual eigenoperators. To this end, let us start by defining $v_{n}={\left[\begin{array}{cc} x & y^{*} \end{array}\right]}^{T}$ as the eigenvectors of **M** ^*T*^, i.e., **M** ^*T*^ ***v*** = *λ* ***v***, so that the associated eigenoperator is

(6) $${\hat{\Psi }}_{n}\left(z\right)=\overset{N}{\underset{m=1}{\sum }}x_{nm}{\hat{a}}_{m}\left(z\right)+\overset{N}{\underset{m=1}{\sum }}y_{nm}^{*}{\hat{a}}_{m}^{†}\left(z\right)$$

and, analogously, the associated noise operator is given by

(7) $${\hat{\zeta }}_{n}\left(z\right)=\overset{N}{\underset{m=1}{\sum }}x_{nm}{\hat{F}}_{m}\left(z\right)+\overset{N}{\underset{m=1}{\sum }}y_{nm}^{*}{\hat{F}}_{m}^{†}\left(z\right)$$

Additionally, given the structure of $\hat{a}\left(z\right)$ in (1), it is always possible to write the evolution matrix **M** as a block matrix whose elements are the matrices **P**, characterizing linear phenomena and coupling between modes, and **Q** accounting for nonlinear effects mixing annihilation and creation operators:

(8) $$M=\left[\begin{array}{cc} P & Q\\ Q^{*} & P^{*} \end{array}\right]$$

Therefore, the eigenvalue problem for the transpose of the evolution matrix reduces to the following conditions:

(9) $$P^{T}x+Q^{†}y^{*}=\lambda x$$

(10) $$Q^{T}x+P^{†}y^{*}=\lambda y^{*}$$

It is crucial to distinguish two cases in the structure of eigenvectors and, consequently, that of their associated operators, depending on whether the eigenvalues are real or complex numbers, i.e., depending on whether we are at the broken or unbroken phase of an NH system.

#### 2.4.1 Real eigenvalues

If λ∈R , by complex conjugating Eq. (10), we obtain **Q** ^†^ x* + **P** ^*T*^ **y** = *λ* **y**, and, by comparing with Eq. (9) we can establish that y* = x*. Therefore, the eigenvectors associated with real eigenvalues exhibit the general structure $v={\left[\begin{array}{cc} x & x^{*} \end{array}\right]}^{T}$ , leading to the eigenoperators:

(11) $${\hat{\Psi }}_{n}\left(z\right)={\hat{\psi }}_{n}\left(z\right)+{\hat{\psi }}_{n}^{†}\left(z\right)$$

where ${\hat{\Psi }}_{n}\left(z\right)$ is explicitely written as a Hermitian operator with ${\hat{\psi }}_{n}\left(z\right)=\overset{N}{\underset{m=1}{\sum }}x_{nm}{\hat{a}}_{m}\left(z\right)$ being a linear combination of physical waveguide mode operators. Then, the spatial evolution of this operator can be computed following Eq. (4).

Furthermore, Eq. (11) reveals that the eigenoperators ${\hat{\Psi }}_{n}\left(z\right)$ , associated with real eigenvalues, correspond to a quadrature operators in the basis of the operators ${\hat{\psi }}_{n}\left(z\right)$ . Thus, this notation reveals that the photon statistics of such eigenoperators can be measured with conventional techniques consisting of: (1) a unitary transformation that changes the basis to another one in which a selected eigenmode of **M** ^*T*^ is a member of the basis, and (2) homodyne detectors to extract the associated photon statistics, as eigenoperators of real eigenvalues represent quadrature components in this new basis (see Figure 1).

#### 2.4.2 Complex eigenvalues

A different eigenvector/eigenvalue structure arises when λ∈C . After complex conjugation of Eqs. (9) and (10) it can be concluded that if *λ* is an eigenvalue with eigenvector $v={\left[\begin{array}{cc} x & y^{*} \end{array}\right]}^{T}$ , then $w={\left[\begin{array}{cc} y & x^{*} \end{array}\right]}^{T}$ is also an eigenvector with eigenvalue λ*. For each pair of complex eigenvalues, the eigenoperator associated with the eigenvalue *λ* is given by

(12) $${\hat{\Psi }}_{n}\left(z\right)={\hat{\psi }}_{nx}\left(z\right)+{\hat{\psi }}_{ny}^{†}\left(z\right)$$

where ${\hat{\psi }}_{nx}\left(z\right)=\overset{N}{\underset{m=1}{\sum }}x_{nm}{\hat{a}}_{m}\left(z\right)$ and ${\hat{\psi }}_{ny}\left(z\right)=\overset{N}{\underset{m=1}{\sum }}y_{nm}{\hat{a}}_{m}\left(z\right)$ , and the eigenoperator associated with λ* is given by ${\hat{\Psi }}_{n}^{†}\left(z\right)$ . In this case, ${\hat{\Psi }}_{n}\left(z\right)$ is not a Hermitian operator. However, the linear combinations ${\hat{\Psi }}_{n}^{†}\left(z\right)+{\hat{\Psi }}_{n}\left(z\right)$ and $i\left({\hat{\Psi }}_{n}^{†}\left(z\right)-{\hat{\Psi }}_{n}\left(z\right)\right)$ are Hermitian operators that could be characterized with the measurement setup depicted in Figure 1.

## 3 Quantum gain and loss models in coupled waveguide systems

Next, we use the described theoretical framework for the analysis of photonic systems composed of two coupled waveguides, one amplifying and one lossy waveguide. We aim to show how the linear or parametric nature of the gain and loss mechanisms influences the behavior of the eigenvalues, the evolution of the eigenoperators, and the photon statistics of the noise generated by the quantum system.

**Figure 2:**
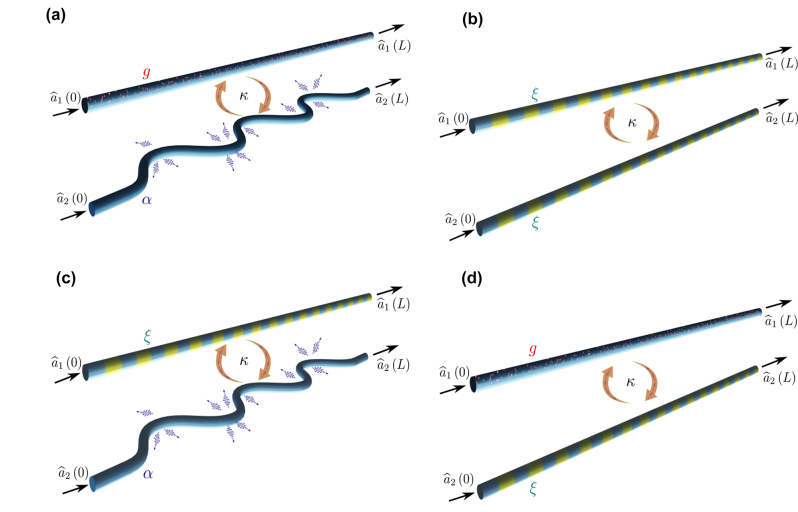
Sketch of coupled waveguides with amplification and loss modeled through a combination of linear and parametric phenomena. In particular, *α* and *g* denote linear loss and gain, respectively, while *ξ* represents nonlinear effects leading to gain or loss depending on the quadrature component considered. Additionally, *κ* represents the coupling strength between waveguides. (a) Linear gain – linear loss. (b) Parametric gain – parametric loss. (c) Parametric gain – linear loss. (d) Linear gain – parametric loss.

To this end, we consider four different scenarios, which are schematically depicted in Figure 2: both gain and loss modeled through linear processes, both modeled through a parametric process, and the two possible combinations of linear and parametric mechanisms. It is important to note that in the figure, linear loss is showcased as arising from waveguide bending for a comprehensible graphical representation. However, the analysis proposed in the manuscript is not specific or restricted to them. Additionally, parametric gain and loss are indistinguishable in the depicted waveguides, as the mechanism underlying both phenomena is the same.

### 3.1 Linear gain-linear loss

As depicted in Figure 2(a), we start by analyzing a linear amplifying waveguide with gain factor *g*, coupled with strength *κ* to a linear lossy waveguide with loss factor *α*. The spatial evolution of the photonics modes in the system is described through the following coupled differential equations:

(13) $$\partial_{z}{\hat{a}}_{1}\left(z\right)=g{\hat{a}}_{1}\left(z\right)+i\kappa {\hat{a}}_{2}\left(z\right)+\sqrt{2g}{\hat{f}}_{1}^{†}\left(z\right)$$

(14) $$\partial_{z}{\hat{a}}_{2}\left(z\right)=-\alpha {\hat{a}}_{2}\left(z\right)+i\kappa {\hat{a}}_{1}\left(z\right)+\sqrt{2\alpha }{\hat{f}}_{2}\left(z\right)$$

where the bosonic noise operators and their associated coefficients are responsible for maintaining the commutation relations. Equivalently, the system’s spatial evolution can be compactly written as in Eq. (1), from where we can straightforwardly identify the matrices **P** and **Q** in the block matrix **M**. As mentioned, linear phenomena do not involve mixing of creation and annihilation operators, and, therefore, **Q** = **0**. In addition, **M** ^*T*^ = **M**, since the coupling between the waveguides is reciprocal. Next, computing eigenvalues and eigenvectors of this system implies to solve Eqs. (9) and (10), which upon substitution of the symmetric matrix **P** and **Q** reduce to **Px** = *λ* **x** and P*y* = *λ* y*, respectively. From these relations, we can predict that if the matrix **P** has eigenvalue *λ* with eigenvector **x**, then the matrix **M** would share the same eigenvalue *λ* with eigenvector ${\left[\begin{array}{cc} x & 0 \end{array}\right]}^{T}$ , and also one eigenvalue λ* with eigenvector ${\left[\begin{array}{cc} 0 & x^{*} \end{array}\right]}^{T}$ , confirming that complex eigenvalues appear on complex conjugate pairs. At the same time, we find that real eigenvalues appear with duplicity 2. Therefore, the *N*-dimensional eigenvectors of the matrix **P** will define the 2*N*-dimensional eigenvectors of the matrix **M**. For the sake of simplicity and to compare to the classical analogue of two coupled waveguides with balanced gain and loss, we consider the particular case where *g* = *α*. Therefore, we obtain two pairs of degenerated eigenvalues (four eigenvalues):

(15) $$\lambda_{1,2\;\pm }=\pm \sqrt{\alpha^{2}-\kappa^{2}}$$

In the notation adopted, the ± sign refers to the positive or negative square root, while the sub-index 1 (2) refers to eigenvalues or eigenvectors resulting from solutions to Eq. (9) (Eq. (10)) in the reduced form described above. Therefore, we have eigenvalues *λ*_1±_ from Eq. (9) and *λ*_2±_ from Eq. (10). The associated normalized eigenvectors are given by

(16) $$v_{1\pm }=\frac{1}{\sqrt{1+{\left|\beta_{\pm }\right|}^{2}}}{\left[\begin{array}{cccc} 1 & i\beta_{\pm } & 0 & 0 \end{array}\right]}^{T}$$

(17) $$v_{2\pm }=\frac{1}{\sqrt{1+{\left|\beta_{\pm }\right|}^{2}}}{\left[\begin{array}{cccc} 0 & 0 & 1 & -i\beta_{\pm } \end{array}\right]}^{T}$$

where $\beta_{\pm }=-\frac{1}{\kappa }\left(-\alpha \pm \sqrt{\alpha^{2}-\kappa^{2}}\right)$ .

**Figure 3:**
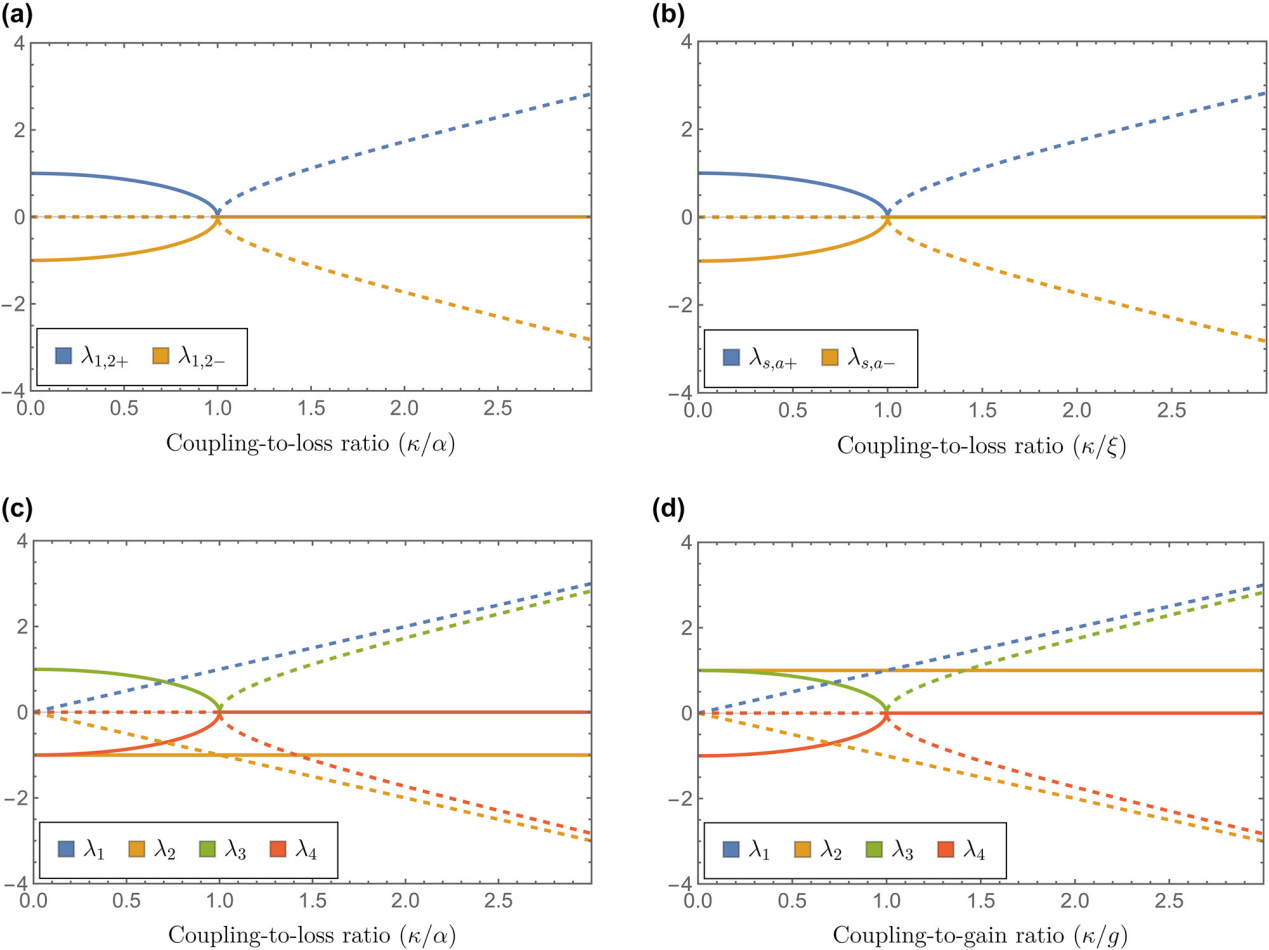
Real (solid lines) and imaginary parts (dashed lines) of the eigenvalues in coupled waveguides with balanced gain and loss modeled through a combination of linear and parametric phenomena. (a) Linear gain – linear loss. (b) Parametric gain – parametric loss. (c) Parametric gain – linear loss. (d) Linear gain – parametric loss.

As noticed from (15), the eigenvalues will be real or complex numbers depending on the interplay between the coupling coefficient *κ* and the loss (gain) coefficient *α*. It is clear from Figure 3(a) that there is a phase-transition point in the parameter space, determined by the coupling-to-loss ratio *κ*/*α* = 1, where all the eigenvalues coalesce and vanish, i.e., *λ*_1,2±_ = 0, establishing the transition from a phase with real eigenvalues to another phase where all the eigenvalues are complex numbers. At this transition point *β*_±_ = 1 and the space expanded by the eigenvectors shrinks because they coalesce in pairs, with $v_{1\pm }=\frac{1}{\sqrt{2}}{\left[\begin{array}{cccc} 1 & i & 0 & 0 \end{array}\right]}^{T}$ and $v_{2\pm }=\frac{1}{\sqrt{2}}{\left[\begin{array}{cccc} 0 & 0 & 1 & -i \end{array}\right]}^{T}$ . At the eigenvalue/eigenvector level, the linear gain/linear loss configuration has the clearest connection with the classical description of the system. From a classical perspective, this phase-transition point corresponds to an exceptional point (EP), where all eigenvalues and eigenvectors coalesce [2]. From the quantum optics perspective, the distinction between creation and destruction operators, doubles the eigenspace dimension, and not all eigenvectors coalesce due to complex conjugation. In addition, the inclusion of noise terms also distinguishes the quantum approach [39].

#### 3.1.1 Eigenvectors and eigenoperators for real eigenvalues

Our analysis focuses on the case of real eigenvalues of **M** leading to amplification and attenuation effects, and how they converge to the phase transition, also leading to gain–loss compensation. As anticipated by Eq. (15) and Figure 3(a), if *α* > *κ* we have that all eigenvalues are real and the eigenvectors can be compactly written as in (16) and (17) ignoring the module operation since *β*_±_ would only take real values. We can verify that in the limit *κ* → 0, the eigenvalues become $\lambda_{1,2\pm }=\pm \sqrt{\alpha^{2}-\kappa^{2}}≃\pm \alpha$ . Additionally, *β*_+_ = 0 and *β*_−_ → ∞, and therefore, we recover the limit case of two uncoupled amplifying and lossy waveguides.

As previously explained, eigenvectors associated with real eigenvalues can be represented in a way that leads to Hermitian eigenoperators describing the system’s modes. As the + and − eigenvectors have the same eigenvalue with multiplicity two, it is possible to write a different basis that complies with the required structure $v={\left[\begin{array}{cc} x & x^{*} \end{array}\right]}^{T}$ by defining two quadratures:

(18) $$v_{X\pm }=\frac{1}{2}\left(v_{1\pm }+v_{2\pm }\right)=\frac{1}{2\sqrt{1+\beta_{\pm }^{2}}}{\left[\begin{array}{cccc} 1 & i\beta_{\pm } & 1 & -i\beta_{\pm } \end{array}\right]}^{T}$$

(19) $$v_{Y\pm }=\frac{i}{2}\left(v_{2\pm }-v_{1\pm }\right)=\frac{1}{2\sqrt{1+\beta_{\pm }^{2}}}{\left[\begin{array}{cccc} -1 & -i\beta_{\pm } & 1 & -i\beta_{\pm } \end{array}\right]}^{T}$$

from where the associated Hermitian eigenoperators ${\hat{\Psi }}_{X\pm }\left(L\right)$ and ${\hat{\Psi }}_{Y\pm }\left(L\right)$ can be written.

#### 3.1.2 Eigenoperators variance

Next we study the noise properties in the system by computing the variance for each quadrature eigenoperator:

(20) $${\left(\Delta \Psi_{n}\left(L\right)\right)}^{2}=\left\langle {\hat{\Psi }}_{n}^{2}\left(L\right)\right\rangle -{\left\langle {\hat{\Psi }}_{n}\left(L\right)\right\rangle }^{2}$$

where the angle brackets  〈 〉 denote expectations values referred to the quantum state of the system, at the beginning of the waveguide. In this manner, the expectation values at the end of the waveguide are written in terms of the expectation values at the beginning of the waveguide. We are interested in the noise inherent to the device, due to the nature of the gain and loss mechanisms and the thermal conditions in the system. Therefore, in all the systems analyzed in this manuscript, the expectation values computed refer to the joint state describing input vacuum states in both waveguides and also the waveguides at a given temperature *T*. Such conditions can be described through the joint density matrix $\hat{\rho }=\hat{\rho_{1}}⊗\hat{\rho_{2}}$ with ${\hat{\rho }}_{1,2}=\left(1-e^{-\hbar \omega /k_{B}T_{1,2}}\right)\underset{n_{f1,2}}{\sum }e^{-n_{f1,2}\hbar \omega /k_{B}T_{1,2}}\left|n_{f1,2}\right\rangle \left\langle n_{f1,2}\right|$ , where *ℏ* is the reduced Planck constant, *k*_*B*_ is the Boltzmann constant and *n*_*fn*_ represents the number of thermal photons in mode *n*. Here, it is important to remark that if the linear loss of the waveguide corresponds to dissipation loss, then the temperature *T* physically corresponds to the temperature of the system. However, for scattering and/or radiation loss, then the temperature *T* physically corresponds to an effective temperature describing the external background noise coupled to the waveguide.

As the eigenoperators involved only contain linear combinations of annihilation and creation operators, the expectation values of the individual operators vanish, and the contribution to the variance is only due to the squared operator expectation value, i.e., ${\left(\Delta \Psi_{n}\left(L\right)\right)}^{2}=\left\langle {\hat{\Psi }}_{n}^{2}\left(L\right)\right\rangle$ . Additionally, it is easy to verify that the + and − eigenoperators in both quadratures present the same statistics ${\left(\Delta \Psi_{Y\pm }\left(L\right)\right)}^{2}={\left(\Delta \Psi_{X\pm }\left(L\right)\right)}^{2}$ , which is reasonable considering the absence of phase-sensitive gain or loss mechanisms that influence differently each quadrature in the system, as it is the case in a parametric process represented through a squeezing transformation. Therefore, we have

(21) $$\begin{array}{ll} {\left(\Delta \Psi_{X,Y\pm }\left(L\right)\right)}^{2} & =\frac{1}{4}e^{2\lambda_{\pm }L}+\frac{\alpha \;\left(e^{2\lambda_{\pm }L}-1\right)}{4\left(1+\beta_{\pm }^{2}\right)\lambda_{\pm }}\\ & \;\times \left(2\left\langle {\hat{n}}_{f1}\right\rangle +2\beta_{\pm }^{2}\left\langle {\hat{n}}_{f2}\right\rangle +1+\beta_{\pm }^{2}\right) \end{array}$$

where ${\hat{n}}_{fn}={\hat{f}}_{n}^{†}\left(0\right)\;{\hat{f}}_{n}\left(0\right)$ represents the number operator for thermal photons in mode *n*. The mean number of photons in a thermal state [43] is given by ${\left\langle \hat{n}\right\rangle }_{T}=\frac{1}{e^{\hbar \omega /k_{B}T}-1}$ . It is easy to distinguish that the first term in Eq. (21) corresponds to the contribution from the photonic part in the eigenoperator, while the second term is contributed by the noise.

**Figure 4:**
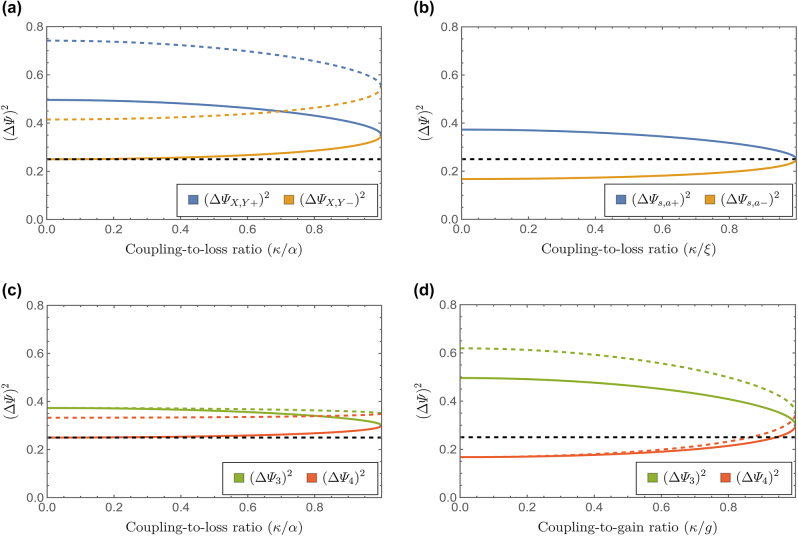
Eigenoperators variance as a function of the coupling-to-loss (gain) ratio, considering input vacuum states and (i) waveguides at temperature *T* = 0 (solid lines), (ii) one thermal photon $\left\langle {\hat{n}}_{f}\right\rangle =1$ in each waveguide (dashed lines). The plots correspond to *α L* = 0.2. The black dashed line denotes the vacuum fluctuations limit. (a) Linear gain – linear loss. (b) Parametric gain – parametric loss. (c) Parametric gain – linear loss. (d) Linear gain – parametric loss.

It is interesting to analyze some limiting cases. For instance, in the zero temperature limit (*T* = 0), where $\left\langle {\hat{n}}_{f1}\right\rangle =\left\langle {\hat{n}}_{f2}\right\rangle =0$ , the variance in Eq. (21) reduces to ${\left(\Delta \Psi_{X,Y\pm }\left(L\right)\right)}_{T0}^{2}=\frac{1}{4}e^{2\lambda_{\pm }L}+\frac{\alpha }{4\lambda_{\pm }}\left(e^{2\lambda_{\pm }L}-1\right)$ . It can be inferred from these equation that, for a given coupling-to-loss ratio within the limits for real eigenvalues (*κ*/*α* ≤ 1), increasing *L* leads to higher variance values, although eigenmodes associated with different eigenvalues are influenced differently. Next, let us analyze the behavior of ${\left(\Delta \Psi_{X,Y\pm }\left(L\right)\right)}_{T0}^{2}$ as a function of the coupling-to-loss ratio *κ*/*α* for a given *α L*, represented by the solid line plots in Figure 4(a). As expected, in the limit *κ* → 0, where *λ*_±_ → ±*α*, we recover the variances associated with uncoupled waveguides, i.e., ${\left(\Delta \Psi_{X,Y+}\left(L\right)\right)}^{2}=\frac{1}{4}\left(2e^{2\alpha L}-1\right)$ for a waveguide with linear gain and ${\left(\Delta \Psi_{X,Y-}\left(L\right)\right)}^{2}=\frac{1}{4}$ for the lossy waveguide. When we move away from this limit, a stronger coupling between photons in different waveguide modes causes the variance of the amplified modes to decrease, while the lossy modes increase their variance, until they balance ${\left(\Delta \Psi_{X,Y\pm }\left(L\right)\right)}_{\kappa =\alpha }^{2}=\frac{1}{4}+\frac{1}{2}\alpha L$ at the phase-transition point where the eigenvalues vanish. At the phase transition, linear gain and loss are perfectly balanced and classical signals are neither amplified nor attenuated. However, it is found that the system introduces additional noise that increases the variance in both quadratures. The larger the loss compensation, the stronger the noise. Remarkably, the generated noise at the phase transition only scales linearly with the length of the waveguide, a much slower trend that the exponential scaling of uncoupled (*κ* → 0) waveguides. In fact, it can be demonstrated that, for sufficiently small values of *αL*, the variance at the phase transition equals the geometric mean of the variances for uncoupled waveguides, according to a first-order approximation in the Taylor series. This property of the generated noise holds even if the waveguides are electrically large. Therefore, although noise is unavoidably generated at the phase-transition point (or gain–loss compensation point), its scaling is much slower than that exhibited by uncoupled lossy and amplifying waveguides.

On the other hand, assuming that both waveguides are at the same temperature *T* ≠ 0, then $\left\langle {\hat{n}}_{f1}\right\rangle =\left\langle {\hat{n}}_{f2}\right\rangle \neq 0$ , and we can straightforwardly identify the thermal noise contribution from the resulting variance, as ${\left(\Delta \Psi_{X,Y\pm }\left(L\right)\right)}_{T}^{2}={\left(\Delta \Psi_{X,Y\pm }\left(L\right)\right)}_{T0}^{2}+\frac{\alpha \left(e^{2\lambda_{\pm }L}-1\right)}{2\lambda_{\pm }}\left\langle {\hat{n}}_{f}\right\rangle$ . Dashed lines in Figure 4(a) depict the computed variance for one thermal photon $\left\langle {\hat{n}}_{f}\right\rangle =1$ in each waveguide. We observe a significant increase of the fluctuations in both modes even for a single thermal photon.

It is important to recall that if *α* = *κ* we obtain that all the four eigenvalues vanish *λ*_1,2±_ = 0. Additionally, *β*_±_ = 1 and there is a coalescence in pairs of the eigenvectors. Computing the variance on the quadrature basis leads to ${\left(\Delta \Psi_{X,Y\pm }\left(L\right)\right)}_{\kappa =\alpha }^{2}=\frac{1}{4}+\frac{1}{2}\alpha L\left(\left\langle {\hat{n}}_{f1}\right\rangle +\left\langle {\hat{n}}_{f2}\right\rangle +1\right)$ , which is the same result we recover from Eq. (21) in the limit *λ*_±_ → 0, pointing towards continuity at the phase-transition point. Therefore, both thermal and quantum noise scale linearly with the length of the waveguide at the phase-transition point.

### 3.2 Parametric gain-parametric loss

The next waveguide system under analysis consist also in one amplifying and one lossy waveguides coupled with strength *κ*, as represented in Figure 2(b). Gain and loss are modeled through the same parametric process: a squeezing transformation with squeezing parameter ξ∈R , which is considered a real quantity for simplicity. For this configuration, the spatial evolution of the photonics modes in the system is given by [43]:

(22) $$\partial_{z}{\hat{a}}_{1}\left(z\right)=\xi {\hat{a}}_{1}^{†}\left(z\right)+i\kappa {\hat{a}}_{2}\left(z\right)$$

(23) $$\partial_{z}{\hat{a}}_{2}\left(z\right)=\xi {\hat{a}}_{2}^{†}\left(z\right)+i\kappa {\hat{a}}_{1}\left(z\right)$$

Again, the system can be equivalently described as in Eq. (1), from where **P** and **Q** matrices can be easily identified.

Using a common mechanism for both gain and loss makes it possible to interchange the role of the amplifying and lossy waveguides, endowing the system with an inherent symmetry. Therefore, a natural basis to analyze the system is the use of symmetric, $v_{s}={\left[\begin{array}{cccc} x & x & y^{*} & y^{*} \end{array}\right]}^{T}$ , and anti-symmetric, $v_{a}={\left[\begin{array}{cccc} x & -x & y^{*} & -y^{*} \end{array}\right]}^{T}$ , eigenvectors. Then, substituting these constraints into Eqs. (9) and (10) we find that both symmetric and anti-symmetric eigenvectors share the same pair of eigenvalues

(24) $$\lambda_{s,a\;\pm }=\pm \sqrt{\xi^{2}-\kappa^{2}}$$

which can be real or complex numbers depending on the interplay between squeezing and coupling parameters. A phase transition occurs at *κ*/*ξ* = 1, where all eigenvalues simultaneously coalesce and vanish *λ*_*s*,*a*±_= 0, as depicted in Figure 3(b). It is clear from Eq. (24) and Figure 3(b) that the eigenvalues behave exactly the same as in the linear gain – linear loss case, with the squeezing parameter *ξ* playing the role of the loss (gain) coefficient *α* in (15). At the same time, the eigenvectors and the generated noise have very different properties.

#### 3.2.1 Eigenvectors and eigenoperators for real eigenvalues

Hereafter, we will focus on the regime where the degenerate eigenvalues are real numbers, i.e., *ξ* ≥ *κ*. For real eigenvalues, the eigenvectors take the form

(25) $$v_{s\pm }=\frac{1}{2}{\text{e}}^{\text{i}\varphi_{s\pm }/2}{\left[\begin{array}{cccc} {\text{e}}^{-\text{i}\varphi_{s\pm }/2} & {\text{e}}^{-\text{i}\varphi_{s\pm }/2} & {\text{e}}^{\text{i}\varphi_{s\pm }/2} & {\text{e}}^{\text{i}\varphi_{s}\pm /2} \end{array}\right]}^{T}$$

(26) $$v_{a\pm }=\frac{1}{2}{\text{e}}^{\text{i}\varphi_{a\pm }/2}{\left[\begin{array}{cccc} {\text{e}}^{-\text{i}\varphi_{a\pm }/2} & -{\text{e}}^{-\text{i}\varphi_{a\pm }/2} & {\text{e}}^{\text{i}\varphi_{a\pm }/2} & -{\text{e}}^{\text{i}\varphi_{a\pm }/2} \end{array}\right]}^{T}$$

where ${\text{e}}^{\text{i}\varphi_{s\pm }}=\frac{\pm \sqrt{\xi^{2}-\kappa^{2}}-i\kappa }{\xi }$ and ${\text{e}}^{\text{i}\varphi_{a\pm }}=\frac{\pm \sqrt{\xi^{2}-\kappa^{2}}+i\kappa }{\xi }$ , and therefore, $\varphi_{a\pm }=\arctan\frac{\kappa }{\pm \sqrt{\xi^{2}-\kappa^{2}}}$ and $\varphi_{s\pm }=\arctan\frac{-\kappa }{\pm \sqrt{\xi^{2}-\kappa^{2}}}$ . The ± sign in the phase angle represents the positive or negative square root in the eigenvalue computation. It is evident that, apart from global phase factors, the eigenvectors in Eqs. (25) and (26) follow the structure predicted for real eigenvalues and associated to Hermitian operators. At the eigenvalues phase-transition point *κ*/*ξ* = 1, we have that ${\text{e}}^{\text{i}\varphi_{s\pm }}=-i$ and ${\text{e}}^{\text{i}\varphi_{a\pm }}=i$ , and the eigenvectors associated to the + and − eigenvalues in each mode coalesce. Therefore, at the phase-transition point where the four eigenvalues vanish *λ*_*s*,*a*±_= 0, the eigenvectors only coalesce in pairs. In this case, the phase sensitivity of the system precludes the existence of an EP where all eigenvalues and eigenvectors coalesce.

Finally, the eigenoperators ${\hat{\Psi }}_{s\pm }\left(L\right)$ and ${\hat{\Psi }}_{a\pm }\left(L\right)$ can be obtained from the knowledge of the symmetric and anti-symmetric eigenvectors, as specified in previous sections.

#### 3.2.2 Eigenoperators variance

Computing the variance of the eigenoperators considering vacuum states in both waveguides and both at the same temperature *T* reveals important aspects of the fluctuations in the coupled system:

(27) $${\left(\Delta \Psi_{s,a\pm }\left(L\right)\right)}_{T}^{2}=\frac{1}{4}e^{2\lambda_{s,a\;\pm }L}$$

First, as expected from the absence of noise sources in the evolution equations, there is no thermal noise contribution to the system variance; therefore, the system is only affected by the unavoidable quantum noise that results from vacuum fluctuations. Second, for both the symmetric and anti-symmetric modes, the variance behaves similarly to the variance in a squeezing transformation. The difference is that instead of twice the squeezing amplitude in the exponential (±2*ξ*) we now have this term replaced by $2\lambda_{s,a\pm }L=\pm 2\sqrt{\xi^{2}-\kappa^{2}}\;L$ , which can be interpreted as a modified squeezing amplitude. It suggests that increasing the coupling strength between the waveguides decreases the degree of squeezing we can obtain, while a larger interaction length would have the opposite effect. At the uncoupled waveguides limit *κ* → 0, the variance reduces to ${\left(\Delta \Psi_{s,a\pm }\left(L\right)\right)}^{2}=\frac{1}{4}e^{\pm 2\xi L}$ . Regarding the dependence of the eigenoperator’s variance on the waveguide length *L*, Eq. (27) anticipates an exponential reduction (increase) of the fluctuations in the squeezed (anti-squeezed) modes, starting from the vacuum value at the infinitesimal waveguide limit.

Figure 4(b) confirms that a pair of eigenmodes exhibit a squeezed variance with a minimum value when *κ* → 0, which increases with the coupling-to-loss ratio *κ*/*ξ* but remains squeezed until it reaches the vacuum fluctuation limit 1/4 at the critical phase-transition point. As for the anti-squeezed modes, their variance reduces from its maximum value for the uncoupled waveguides limit until it also converges to the vacuum fluctuation limit 1/4 at the phase-transition point. As explained, the evolution of the eigenmodes and their variances are not affected by the system’s temperature. Therefore, in the presence of thermal photons, the variance of the eigenmodes coincides with the variance at *T* = 0. At the transition point, gain and loss are perfectly compensated, and their squeezing is nil. At the same time, such gain loss compensation occurs without any noise amplification. Thus, the variance experienced by the photonic modes at the phase-transition point correspond to unamplified vacuum fluctuations.

### 3.3 Parametric gain-linear loss

After analyzing systems of two coupled waveguides exhibiting both linear gain and loss or both parametric gain and loss, a natural question arises about the effect of combining linear and nonlinear phenomena. Therefore, we study a system composed by an amplifying waveguide, with parametric gain modeled through a squeezing transformation with squeezing parameter *ξ*, coupled with strength *κ* to a linear lossy waveguide with loss factor *α*, as depicted in Figure 2(c). The spatial evolution of the photonic modes in the system can be written as follows:

(28) $$\partial_{z}{\hat{a}}_{1}\left(z\right)=\xi {\hat{a}}_{1}^{†}\left(z\right)+i\kappa {\hat{a}}_{2}\left(z\right)$$

(29) $$\partial_{z}{\hat{a}}_{2}\left(z\right)=-\alpha {\hat{a}}_{2}\left(z\right)+i\kappa {\hat{a}}_{1}\left(z\right)+\sqrt{2\alpha }{\hat{f}}_{2}\left(z\right)$$

from where the matrices **M**, **P** and **Q** can be identified. Then, from Eqs. (9) and (10), the eigenvalues for the system with balanced gain and loss *α* = *ξ* can be obtained:

(30) $$\lambda_{1,2}=-\alpha \pm i\kappa$$

(31) $$\lambda_{3,4}=\pm \sqrt{\alpha^{2}-\kappa^{2}}$$

From the computed eigenvalues and their dependence on the coupling-to-loss ratio depicted in Figure 3(c), we note that real-valued *λ*_1,2_ will not occur for the balanced system under analysis; otherwise, there should be no coupling between waveguides. In fact, the eigenvalues in (30) coincide with those in a linear loss-loss configuration. The other pair of eigenvalues *λ*_3,4_ coincides with the eigenvalues for the cases of linear gain – linear loss and parametric gain – parametric loss previously addressed. Therefore, *λ*_3,4_ might take real or complex values depending on the interplay between loss (or gain) and coupling strength, with $\lambda_{3,4}\in C$ for *α* < *κ*, while $\lambda_{3,4}\in R$ for *α* ≥ *κ*, being *α* = *κ* the phase-transition point (*λ*_3,4_ = 0). Thus, we find that different classes of gain and loss can compensate each other, and they follow the same eigenvalue structure.

#### 3.3.1 Eigenvectors and eigenoperators for real eigenvalues

As we are interested in gain–loss configurations, we focus on the pair of eigenvalues *λ*_3,4_. The associated normalized eigenvectors for *α* ≥ *κ* can be compactly written as follows:

(32) $$v_{3,4}=\frac{1}{\sqrt{1+A_{3,4}^{2}}}{\left[\begin{array}{cccc} -iA_{3,4}, & 1, & iA_{3,4}, & 1 \end{array}\right]}^{T}$$

with $A_{3,4}=\frac{\alpha \pm \sqrt{\alpha^{2}-\kappa^{2}}}{\kappa }$ , from which the corresponding eigenoperators ${\hat{\Psi }}_{3,4}\left(L\right)$ can be obtained.

#### 3.3.2 Eigenoperators variance

The variance characterizing the modes in the output of the waveguides can be computed as follows:

(33) $$\begin{array}{ll} {\left(\Delta \Psi_{3,4}\left(L\right)\right)}_{T}^{2} & =\frac{1}{4}e^{2\lambda_{3,4}L}+\frac{1}{\left(1+A_{3,4}^{2}\right)}\frac{\alpha }{4\lambda_{3,4}}\;\\ & \;\times \left(e^{2\lambda_{3,4}L}-1\right)\left(1+\left\langle {\hat{n}}_{f2}\right\rangle \right) \end{array}$$

from which it is clear that the fluctuations in the system would be the result of the combined action of quantum and thermal noise, the latter contributed by the linear lossy waveguide. In the limit case of waveguides at zero temperature *T* = 0, where no thermal photons populate the waveguides and therefore their expectation value vanish $\left\langle {\hat{n}}_{f2}\right\rangle =0$ , the variance reduces to ${\left(\Delta \Psi_{3,4}\left(L\right)\right)}_{T0}^{2}=\frac{1}{4}e^{2\lambda_{3,4}L}+\frac{1}{\left(1+A_{3,4}^{2}\right)}\frac{\alpha }{4\lambda_{3,4}}\;\left(e^{2\lambda_{3,4}L}-1\right)$ . Again, in the uncoupled waveguides limit *κ* → 0, where *λ*_3,4_ → ±*α*, we recover the signature variances: (i) ${\left(\Delta \Psi_{3}\left(L\right)\right)}^{2}=\frac{1}{4}e^{2\alpha L}$ for the waveguide with parametric gain and (ii) ${\left(\Delta \Psi_{4}\left(L\right)\right)}^{2}=\frac{1}{4}$ for a linear lossy waveguide. Solid line plots in Figure 4(c) confirm that at *T* = 0 none of the Hermitian eigenoperators exhibits a variance squeezed below the vacuum limit $\frac{1}{4}$ , independently of the coupling-to-loss (gain) ratio. As expected, the maximum and minimum fluctuations correspond to the uncoupled case.

These values are modified by the coupling strength between the waveguides until they balance at the corresponding phase-transition point, i.e., ${\left(\Delta \Psi_{3,4}\left(L\right)\right)}^{2}$ = $\frac{1}{4}+\frac{1}{4}\alpha L$ . Similar to the linear loss-linear gain case, it is found that the scaling of the noise variance is linear with the length of the waveguide *αL*, which is a much slower scaling that the exponential trend of the uncoupled waveguides. Therefore, this property is found to be insensitive to the specific gain/loss model. Moreover, at the phase-transition point, for sufficiently small values of *αL*, it is found again that such linear scaling corresponds to the geometric mean of the variances for uncoupled waveguides, according to a first-order approximation in the Taylor series. That is to say, the result corresponds to taking the first-order term of the Taylor series of the variances for the two uncoupled waveguides, and then taking the geometric mean of those two values. Finally, we note that the variance value at the phase transition is smaller than its equivalent in the linear gain/linear loss system, demonstrating that parametric amplification can compensate linear loss with a smaller noise production, albeit at the cost of being phase sensitive.

Dashed lines in Figure 4(c) depict the variance behavior in the presence of thermal photons, for $\left\langle {\hat{n}}_{f2}\right\rangle =1$ . We observe that ${\left(\Delta \Psi_{4}\right)}^{2}$ is more sensitive to the change in the thermal population, increasing its variance in the limit *κ* → 0 compared to its value in the absence of thermal photons, as expected for a mode representing linear loss. On the other hand, the value of ${\left(\Delta \Psi_{4}\right)}^{2}$ when *κ* → 0 does not depend on the thermal population as expected for an individual waveguide with parametric gain. Still, the overall behavior in both variances is similar to the already described for *T* = 0.

As for the variance dependence on the waveguide length, no squeezing will be obtained independently of the value of *L*. In general, for the balanced system squeezing cannot be measured, although it is possible in non-balanced configurations where the eigenoperators associated to the other pair of eigenvalues are allowed to take real values.

### 3.4 Linear gain-parametric loss

Finally, we study the remaining configuration combining linear and parametric gain and loss models, depicted in Figure 2(d). The system consists of a linear amplifying waveguide with gain *g*, coupled with strength *κ* to a lossy waveguide with parametric loss modeled through a squeezing transformation with squeezing parameter *ξ*, as described by the coupled evolution equations:

(34) $$\partial_{z}{\hat{a}}_{1}\left(z\right)=g{\hat{a}}_{1}\left(z\right)+i\kappa {\hat{a}}_{2}\left(z\right)+\sqrt{2g}{\hat{f}}_{1}^{†}\left(z\right)$$

(35) $$\partial_{z}{\hat{a}}_{2}\left(z\right)=\xi {\hat{a}}_{2}^{†}\left(z\right)+i\kappa {\hat{a}}_{1}\left(z\right)$$

The eigenvalues for the specific case of balanced gain and loss *g* = *ξ* are given by

(36) $$\lambda_{1,2}=g\pm i\kappa$$

(37) $$\lambda_{3,4}=\pm \sqrt{g^{2}-\kappa^{2}}$$

Comparing with the eigenvalues in the combined parametric gain – linear loss system, we observe a clear analogy, where *g* plays the role of −*α* in (31). Therefore, a similar analysis and conclusions apply. For example, balanced gain and loss forbids the occurrence of real-valued *λ*_1,2_ except for uncoupled waveguides, a situation that is not relevant to this study. Also, the pair of eigenvalues *λ*_3,4_ exhibits a phase-transition point in parameter space at *κ*/*g* = 1, changing from real to complex values, as depicted in Figure 3(d).

#### 3.4.1 Eigenvectors and eigenoperators for real eigenvalues

The associated eigenvectors in the *g* ≥ *κ* regime are given by

(38) $$v_{3,4}=\frac{1}{\sqrt{A_{3,4}^{2}+1}}{\left[\begin{array}{c} iA_{3,4},1,-iA_{3,4},1 \end{array}\right]}^{T}$$

where $A_{3,4}=\frac{\kappa }{-g\pm \sqrt{g^{2}-\kappa^{2}}}$ . Then, the Hermitian eigenoperators ${\hat{\Psi }}_{3,4}\left(L\right)$ can be immediately derived from the eigenvectors above. Again, at the eigenvalues’ phase-transition point the eigenvectors coalesce, with *A*_3_ = *A*_4_.

#### 3.4.2 Eigenoperators variance

The variance that results from the real eigenvalues can be computed as follows:

(39) $$\begin{array}{ll} {\left(\Delta \Psi_{3,4}\left(L\right)\right)}_{T}^{2} & =\frac{1}{4}e^{2\lambda_{3,4}L}+\frac{A_{3,4}^{2}}{1+A_{3,4}^{2}}\frac{g}{4\lambda_{3,4}}\\ & \;\times \left(e^{2\lambda_{3,4}L}-1\right)\left(1+\left\langle {\hat{n}}_{f1}\right\rangle \right) \end{array}$$

where as in all the previous configurations with linear processes involved, the fluctuations in the system result from the combined action of quantum and thermal noise. As expected, the thermal noise contribution originates from the linear amplifying waveguide. In the absence of thermal photons, i.e., $\left\langle {\hat{n}}_{f1}\right\rangle =0$ for *T* = 0, the variance reduces to ${\left(\Delta {\hat{\Psi }}_{3,4}\left(L\right)\right)}_{T0}^{2}=\frac{1}{4}e^{2\lambda_{3,4}L}+\frac{A_{3,4}^{2}}{\left(1+A_{3,4}^{2}\right)}\frac{g}{4\lambda_{3,4}}\left(e^{2\lambda_{3,4}L}-1\right)$ . From Figure 4(d), we can observe that at *T* = 0 (solid lines) one of the eigenmodes exhibits fluctuations squeezed below the vacuum fluctuation limit. These results can be explained considering the maximum and minimum variances that can be obtained, and that corresponds to the limit *κ* → 0 of uncoupled waveguides, where *λ*_3,4_ → ±*g*. The maximum value of the fluctuations corresponds to ${\left(\Delta \Psi_{3}\left(L\right)\right)}^{2}=\frac{1}{4}\left(2e^{2\mathrm{gL}}-1\right)$ characteristic of a waveguide with linear gain, while the minimum is ${\left(\Delta \Psi_{4}\left(L\right)\right)}^{2}=\frac{1}{4}e^{-2gL}$ signature of the squeezed quadrature in a waveguide with parametric loss.

The most obvious difference with a system where both gain and loss are described through squeezing transformation is that squeezing is not preserved in the whole region where the eigenvalues are real. Therefore, at the phase-transition point, where squeezed and anti-squeezed variances converge, we have that ${\left(\Delta \Psi_{3,4}\left(L\right)\right)}^{2}$ = $\frac{1}{4}+\frac{1}{4}\alpha L$ and the fluctuations are above the quantum vacuum limit 1/4. This can be understood by considering that to obtain a target amplification level, linear gain introduces more noise than its parametric counterpart; therefore, at the compensation point, the fluctuations for the configuration under study exceed those for coupled waveguides featuring parametric gain and loss. As in the previous configuration, at the compensation point, the scaling of the fluctuations is linear with *αL*, with the associated potential benefits. Again, we observe that gain and loss mechanisms of different nature can compensate each other. Also, at the compensation point, coinciding with the phase transition, the variances equal the geometric mean of those at the *κ* → 0 limit for sufficiently small values of *αL*, showing that these conclusions are insensitive with respect to the model of gain/loss. It is important to highlight that, different from the parametric gain – linear loss case, we do get squeezed fluctuations for the system with balanced gain and loss. For thermal population, as depicted by the dashed lines in Figure 4(d) for the specific case of one thermal photon in the system, i.e., $\left\langle {\hat{n}}_{f1}\right\rangle =1$ , we observe that the coupling-to-gain (loss) ratio *κ*/*g* where the squeezed mode crosses the vacuum fluctuation limit is slightly reduced. The influence on the anti-squeezed variance is more evident, considering its value in the limit of infinitesimal waveguides.

Analyzed in terms of its dependence on the waveguide length *L*, the analytical result in (39) predicts an exponential decay of the squeezed mode fluctuations with increasing waveguide length, being in theory infinitesimal for sufficiently large waveguides, and an exponential scaling in the fluctuations for the remaining mode.

## 4 Conclusions

We discussed how different gain and loss mechanisms influence the noise produced in gain–loss compensated coupled photonic waveguides. First, we identified a unitary transformation that reveals the quantum eigenmodes of the spatial evolution so that the associated eigenoperators also correspond with measurable photon statistics. In this way, we could separate the light and quantum noise related to individual eigenoperators even for nondiagonalizable evolution matrices, although arbitrary output light cannot be described through those eigenoperators.

Then, we applied the proposed theoretical method to studying the aforementioned coupled systems. Our results highlight universal properties independent of the gain and loss model, such as a phase transition in the eigenvalue structure, where all eigenvalues vanish and eigenvectors only coalesce in pairs. Simultaneously, some particularities arise. For instance, when only linear effects are present, the phase-transition point corresponds to an exceptional point (EP) in a classical non-Hermitian (NH) system. However, a quantum treatment prevents the coalescence of all eigenvectors as the eigenspace dimension doubles and noise terms are included. On the other hand, when only parametric phenomena are considered, the system’s phase sensitivity impedes the existence of global EPs where all four eigenvectors coalesce, similarly to what occurs in a classical treatment of the system. Our analysis also reveals that gain and loss mechanisms of different nature can compensate each other, inheriting the phase sensitivity of the fluctuations in the parametric process and leading to squeezed fluctuations when the parametric loss is compensated through linear amplification. In other words, our findings suggest that by using quantum resources, in particular squeezing, we can compensate for linear gain or loss while achieving reduced fluctuations compared to solely relying on classical resources. When linear phenomena are involved, the fluctuations result from combined thermal and quantum noise, unlike when only parametric effects are present, and thus, the noise generated is exclusively quantum and corresponds to unamplified vacuum fluctuations. The gain–loss compensation and the phase-transition points coincide for every analyzed waveguide configuration. Interestingly, although noise is unavoidably generated at these phase-transition points, its scaling is linear with the waveguide length and outperforms the exponential scaling exhibited by uncoupled waveguides. It is important to emphasize that although the analysis here focuses on systems with equal gain and loss coefficients, the methods proposed are not restricted to them and can also be applied to the most general scenarios.

We believe these results contribute to deepening our understanding of NH quantum systems and the nontrivial properties at their phase-transition points, with impact on fundamental research and potential applications in gain–loss compensation systems and electrically large photonic networks with reduced fluctuations.
